# Marching Toward Hair Research: HAIRINDIA 2010

**DOI:** 10.4103/0974-7753.58548

**Published:** 2009

**Authors:** Patrick Yesudian

**Affiliations:** President, The Hair Research Society of India, No. 10, Ritherdon Avenue, Vepery, Chennai - 600 007, India. Email: patnirmu@gmail.com


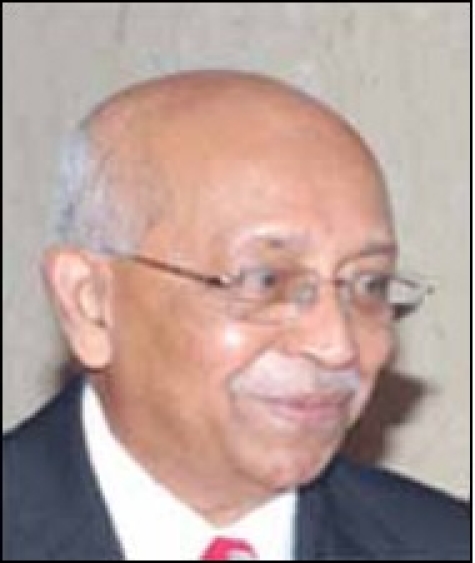


The first issue of the *International of Trichology* (IJT) was officially released on the 14^th^ of June, 2009, by the Principal Secretary of the state for Health and Family Welfare at Chennai, India, where the first seed of hair research was planted in 2004, nearly two decades after the first meeting of the European Hair Research Society was constituted in 1989. The IJT was launched after much deliberation to cater to the need for a separate and exclusive journal dealing with the biology and disorders of this important appendage of the skin-the hair.

After the recent, exciting wo rk on hair biology, particularly the hair follicle stem cell and its potential ability to differentiate into neurons, glia, keratinocytes, smooth muscle cells and melanocytes, all the medical specialities are evincing great interest in trichology. It seems as though the hair follicle stem cell is the future cell in regenerative medicine.

Every brand needs brand recognition else it will not be viable. We, the dermatologists, should be ambassadors for our speciality of trichology. Unfortunately, this superspeciality of dermatology is being usurped by unqualified persons and quacks, more so in the subcontinent. As pointed out by Shyam Verma in our first issue of IJT, the term "trichology" is gaining bad press because of its misuse by non-dermatologists, often the so-called beauticians, cosmetologists and aestheticians. As trichology by definition is the science of hair and scalp disorders, and considering the systemic association of hair and scalp disorders, only a qualified dermatologist can be a trichologist. We, the dermatologists, should admit the bitter truth that this speciality branch of dermatology was often neglected by us in the past, leading way to the entry of quackery mushrooming everywhere. It is time for us to dispel the myths and misconceptions about hair and its disorders and to increase the awareness toward scientific approaches to the correct diagnosis and appropriate management of hair disorders by dermatologists. This is the ultimate goal to be achieved by the Hair Research Society of India.

As yet another step forward toward this goal, we propose to hold the first International Congress of Trichology by the Hair Research Society of India on September 3^rd^, 4^th^ and 5^th^ of the year 2010 at Chennai, a southern historical city of India and the birth place of the Hair Research Society of India. The congress will cover all aspects of trichology and the sessions will be addressed by eminent trichologists from all around the world. The editorial board cordially invites all readers of the IJT to attend the congress in large numbers and make this maiden venture a great success. This would enable us to put the Hair Research Society of India on the map of the trichological societies of the world.

We therefore request readers to mark these three days in their academic calendar for the year 2010. You can be assured of a trichological feast as well as memorable visits to the shore temple of Mamallapuram, which is listed as a UNESCO world heritage site. We eagerly look forward to meeting you in Chennai.

